# Research on Six-Axis Sensor-Based Step-Counting Algorithm for Grazing Sheep

**DOI:** 10.3390/s23135831

**Published:** 2023-06-22

**Authors:** Chengxiang Jiang, Jingwei Qi, Tianci Hu, Xin Wang, Tao Bai, Leifeng Guo, Ruirui Yan

**Affiliations:** 1 College of Computer and Information Engineering, Xinjiang Agricultural University, Urumqi 830052, China; 320213572@xjau.edu.cn (C.J.); 320213558@xjau.edu.cn (T.H.); bt@xjau.edu.cn (T.B.); 2 Agricultural Information Institute of Chinese Academy of Agricultural Sciences, Beijing 100081, China; 82101181269@caas.cn; 3 College of Animal Sciences, Inner Mongolia Agricultural University, Hohhot 010018, China; qijingwei_66@126.com; 4 Xinjiang Agricultural Information Technology Research Centre, Urumqi 830052, China; 5 Ministry of Education Engineering Research Centre for Intelligent Agriculture, Urumqi 830052, China; 6 Xinjiang Wool and Cashmere Engineering Technology Research Center, Urumqi 830099, China; 7 State Key Laboratory of Efficient Utilization of Arid and Semi-Arid Arable Land in North China (Institute of Agricultural Resources and Regional Planning, Chinese Academy of Agricultural Sciences), Beijing 100081, China

**Keywords:** grazing sheep, window peak detection, behavior classification, step counting

## Abstract

Step counting is an effective method to assess the activity level of grazing sheep. However, existing step-counting algorithms have limited adaptability to sheep walking patterns and fail to eliminate false step counts caused by abnormal behaviors. Therefore, this study proposed a step-counting algorithm based on behavior classification designed explicitly for grazing sheep. The algorithm utilized regional peak detection and peak-to-valley difference detection to identify running and leg-shaking behaviors in sheep. It distinguished leg shaking from brisk walking behaviors through variance feature analysis. Based on the recognition results, different step-counting strategies were employed. When running behavior was detected, the algorithm divided the sampling window by the baseline step frequency and multiplied it by a scaling factor to accurately calculate the number of steps for running. No step counting was performed for leg-shaking behavior. For other behaviors, such as slow and brisk walking, a window peak detection algorithm was used for step counting. Experimental results demonstrate a significant improvement in the accuracy of the proposed algorithm compared to the peak detection-based method. In addition, the experimental results demonstrated that the average calculation error of the proposed algorithm in this study was 6.244%, while the average error of the peak detection-based step-counting algorithm was 17.556%. This indicates a significant improvement in the accuracy of the proposed algorithm compared to the peak detection method.

## 1. Introduction

Animal welfare is increasingly gaining priority in animal research, and the timely monitoring of animal behavior daily is a crucial aspect of ensuring their wellbeing. However, manual monitoring of animal behavior poses challenges due to the large animal populations and the dispersion of their activity areas [1]. As a result, the transition from manual monitoring to sensor-based approaches is gradually taking place. By utilizing sensors, it becomes possible to monitor animal behavior more efficiently, thereby expediting the assessment of their current state and ultimately enhancing animal welfare. Among the various sensor options, pedometers became widely employed in modern animal husbandry. These devices provide valuable information regarding the number of steps taken by an animal, serving as an indicator of its activity level. By continuously monitoring animal activity through pedometers in real-time, potential issues such as diseases or lameness can be detected earlier, increasing the chances of timely treatment.

Improving the accuracy and robustness of step-counting algorithms by addressing false step-counting caused by incorrect walking is a crucial approach [2]. Previous research on step-counting algorithms mainly focused on analyzing human and cattle gaits, with limited attention given to algorithms specifically designed for sheep. Therefore, in developing new algorithms for sheep, we refer to existing algorithms from other fields and make improvements and optimizations accordingly. For example, Xiaomin Kang utilized gyroscope data and extracted key walking features in the frequency domain to achieve a walk detection accuracy of 93.76%. Step-counting was performed indirectly by multiplying the walking frequency by the walking duration to mitigate the negative impact of random noise in the time domain. This resulted in a final step counting accuracy of 95.74% [3]. Agata Brajdic evaluated common walk detection and step-counting algorithms for smartphone sensor data. The results showed an error rate of less than 3% for walk detection using standard deviation threshold and step-counting using window peak detection [4]. Sampath Jayalath employed techniques such as engineering noise removal, over-zeroing, and thresholding of single-axis gyroscope data for step counting. The overall accuracy of the step-counting algorithm exceeded 94% [5]. Fuqiang Gu proposed a step-counting algorithm based on peak detection and step feature analysis, specifically targeting over-counting issues caused by erroneous walks. This algorithm outperformed commonly used peak detection methods, improving step-counting accuracy by 6.56% in normal walking, 9.54% in free walking, and 58.92% in erroneous walking scenarios [2]. Claudia Arcidiacono enhanced the accuracy of step counting by using a threshold-based algorithm to calculate the number of steps per cow based on accelerometer data, achieving a total error rate of 9.5% [6].

A growing focus was on classification using acceleration in sheep gait analysis. These studies primarily investigated sheep behaviors such as standing, lying, and walking, but recognition accuracy reached a specific bottleneck. Consequently, researchers started exploring other walking behaviors in sheep, such as lameness, and experimenting with different sampling frequencies and wearing positions to improve recognition accuracy. For instance, F.A.P. Alvarenga utilized a triaxial accelerometer to identify various behaviors of sheep in pastures and employed a decision tree algorithm for classification, including behaviors like grazing, lying, running, standing, and walking [7]. V. Giovanetti conducted tests under grazing conditions to identify sheep behavioral activities (grazing and resting) and achieved a 95% accuracy in recognizing grazing behavior and 94% accuracy in recognizing resting behavior [8]. M. Radeski and V. Ilieski designed an optimized algorithmic model for classifying gait (walking, trotting, and cantering) and posture (standing and lying) using a triaxial accelerometer mounted on the hind legs of sheep to record acceleration values [9]. Jamie Barwick deployed triaxial accelerometers at sheep’s ear, neck, and leg locations to identify lameness movements, achieving prediction accuracies of 82%, 35%, and 87% for the three locations, respectively. Using a moving window classification model, these sensors also classified sheep behaviors such as grazing, standing, walking, and lying. The ear sensor exhibited the highest accuracy (86–95%), followed by neck collar sensors (67–88%) and leg sensors (48–94%) [10,11]. Emily Walton classified sheep’s lying, standing, and walking behaviors using sensors placed on the sheep’s ears and collar. The classifier’s performance was evaluated using sampling frequencies of 8, 16, and 32 Hz, with the best performance achieved at a sampling frequency of 32 Hz [12].

Behaviors such as running, fast walking, and slow walking have some influence on the accuracy of step counting. Most past studies classified these behaviors, while few studies were conducted on some abnormal behaviors, such as sheep shaking their legs and cows flicking their legs, which are not episodic behaviors, and their excessive occurrence can significantly reduce the accuracy of step counting. Since most animal behaviors are unique, it is unrealistic to apply some pedometer algorithm to calculate the step count of a specific animal. This study was based on sheep behavior recognition for step counting to improve the accuracy of sheep step counting. We used six-axis sensors mounted on sheep’s legs to classify sheep’s behaviors, such as slow walking, fast walking, running, and leg shaking. We matched the corresponding algorithm for step counting based on the classification results.

## 2. Materials and Methods

### 2.1. Experimental Site

**Figure 1:**
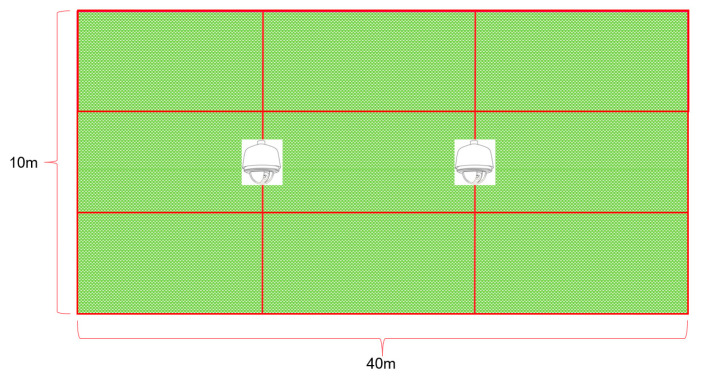
Schematic diagram of the experimental site.

This study was conducted in the small town of Xertala, Inner Mongolia (longitude: 120.00435, latitude: 49.34809), from 22 August 2022 to 19 September 2022. The data collection experiment was carried out in a 40 m × 10 m experimental grassland area, as shown in Figure 1. The experimental grassland was divided into three 40 m × 3.3 m experimental areas, each equipped with natural grass and drinking basins, where the sheep were free to feed and drink during the experiment. In natural grasslands, the primary grass species was sheep grass. To mitigate any detrimental effects of excessively tall grass on sheep behavior, the grass was routinely trimmed to a height of approximately 8 cm.

Two movable fences were installed to control the size of the experimental grassland based on the number of sheep and to facilitate the replacement of the experimental field according to the current availability of edible grass. Surveillance cameras were installed on both sides of the experimental field to record sheep behavior during the experiments. In addition, handheld cameras were also used for multi-angle video recording. The recorded video data primarily served for subsequent sheep behavior analysis and manual step counting. The surveillance cameras and wearable devices shared the same network timestamp, which ensured that video data and accelerometer data remained synchronized in both behavior recognition and manual step counting. To ensure accuracy, the timestamp of the handheld device needed to be calibrated with the network time before the experiment starts, and a secondary calibration using the accelerometer data timestamp was required for subsequent behavior analysis.

### 2.2. Experimental Equipment and Data Acquisition

**Figure 2:**
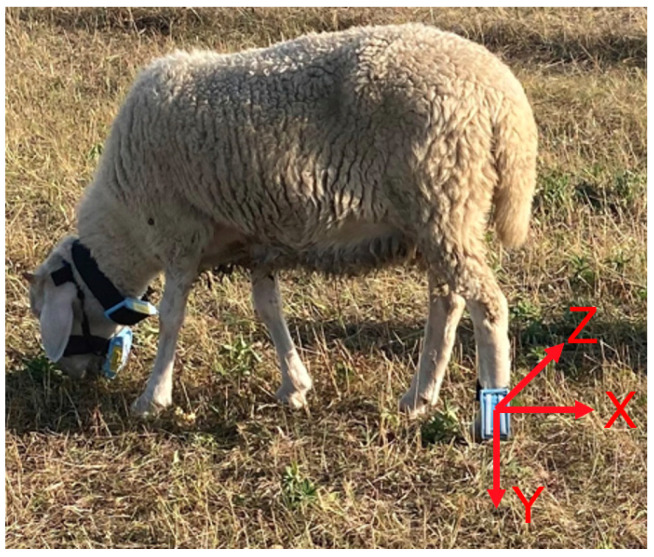
Sheep wearing equipment acceleration fraction.

In this study, ten sheep were observed, and acceleration data were collected by a wearable device worn on the sheep’s legs. The device had an overall weight of 48 g and dimensions of 8.5 cm × 4.7 cm × 2 cm. To prevent any leg abrasions caused by sheep wearing the device, we primarily utilized Velcro made from fabric material for secure attachment. Furthermore, all sharp edges of the device casing were rounded to ensure the safety of the sheep. The wearable device mainly consisted of an ESP8266 module and an MPU6050 six-axis sensor, where the ESP8266 module contained the central control unit and the WIFI data transmission unit. The MPU6050 sensor can collect three-axis acceleration and three-axis angular velocity. The acceleration directions are illustrated in Figure 2, where the x, y, and z axes represent lateral, vertical, and horizontal motion, respectively [13]. The three-axis acceleration values ranged from ±8 g (m/s^2^), while the triaxial angular velocity values ranged from ±500 dps (°/s). In accelerometer-based animal behavior studies, the most used sensor sampling frequencies were 10, 16, and 32 Hz [8]. Therefore, to ensure an accurate classification of the studied behaviors in this study, the sensor’s acquisition frequency was set to 32 Hz [12,14].

### 2.3. Technology Line

**Figure 3:**
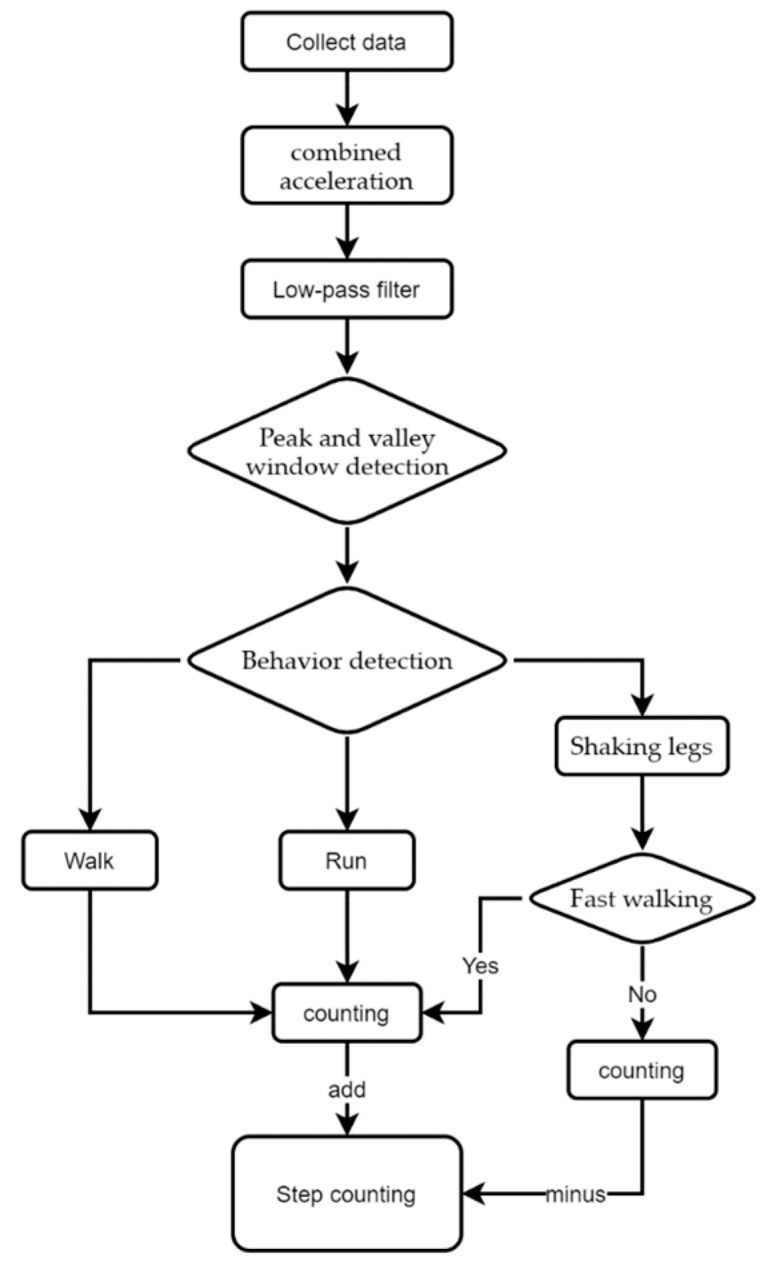
Flowchart for step counting.

Figure 3 shows this paper’s flow chart of the step-counting algorithm. The final total step count = peak count − running behavior window peak count + running steps − leg shaking steps. It should be noted that when calculating the total number of steps, the peak count includes the inaccurate data of running as well as leg shaking behaviors, so when calculating the number of steps of running behaviors, the resulting inaccurate steps need to be subtracted from the number of steps calculated by the peak and valley values.

### 2.4. Data Processing

**Figure 4:**
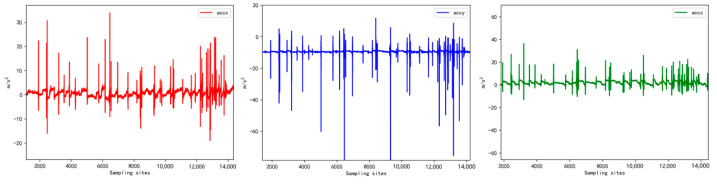
Tri-axis acceleration raw data waveform.

**Figure 5:**
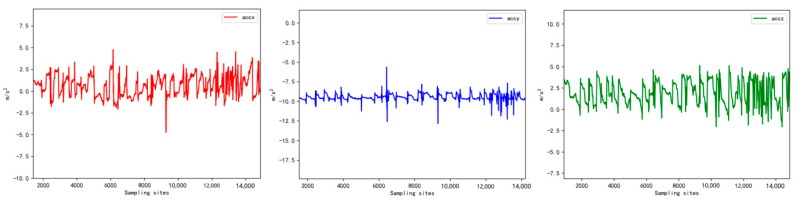
Waveform of triaxial acceleration after filtering.

Due to the high sensitivity of the motion sensor, it tended to capture subtle noise fluctuations generated by sheep movement during walking. To address this issue, the collected raw data needed to be filtered. Figure 4 illustrates the waveform of the raw data obtained from the device’s three-axis acceleration readings. The noise fluctuations present in the data primarily consisted of high-frequency signals. To mitigate this noise, a low-pass filtering technique was employed [15]. The results of this filtering process are presented in Figure 5.

In the experiment, the utilization of single-axis acceleration for step counting may introduce inaccuracies due to changes in the device’s orientation caused by the sheep’s movement. Furthermore, analyzing the three-axis acceleration data before and after filtering revealed that the acceleration data from individual axes did not sufficiently capture the characteristics of sheep walking behavior, relying solely on this data was inadequate for accurately determining the number of steps. As a result, this paper adopted the approach of using combined acceleration to accurately calculate the final step count [16,17,18]. The formula for calculating the combined acceleration is presented in Equation (1).

(1) $$acc=\sqrt{{accx}^{2}+{accy}^{2}+{accz}^{2}}$$

**Figure 6:**
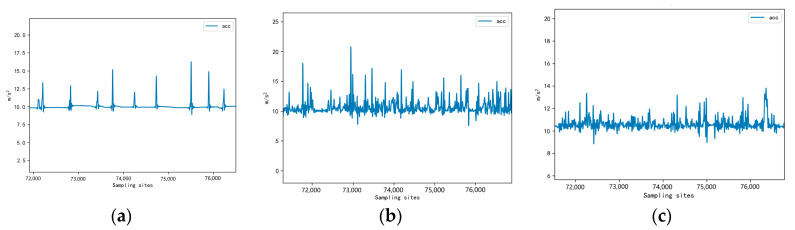
Combined acceleration waveform after filtering. (**a**) Leg data waveform; (**b**) Jaw data waveform; (**c**) Neck data waveform.

After calculating the combined acceleration, the data were processed by low-pass filtering, and the processed data, in terms of the waveform, could better reflect the characteristics of sheep walking behavior and eliminate the interference of equipment direction change on the data. The waveform of the completed data is shown in Figure 6.

By comparing the data waveforms of the neck, jaw, and leg sensors in Figure 6, it was evident that the leg sensors provided a more accurate representation of walking movements during grazing and exercise [19]. Therefore, the leg data were mainly collected for analysis in this article, and a total of 672 MB of leg sensor data were acquired through the wearable device sensors. The experimental data used in this study are shown in Table 1.

### 2.5. Peak and Valley Window Detection

During sheep walking, the raw data collected by the wearable device exhibited a certain periodicity in the waveform after data processing. Leveraging this characteristic, the number of steps can be calculated using the peak detection method [20,21,22]. A peak detection window with a window size of *n* was established to eliminate the interference of abnormal pseudo-peaks on step counting. This was carried out by considering *n* data points to the left and right of the waveform, as described in Equation (2). Here, *acc_i_* represents the acceleration value at the current moment, and A and B values are used for determining wave peaks.

**Figure 7:**
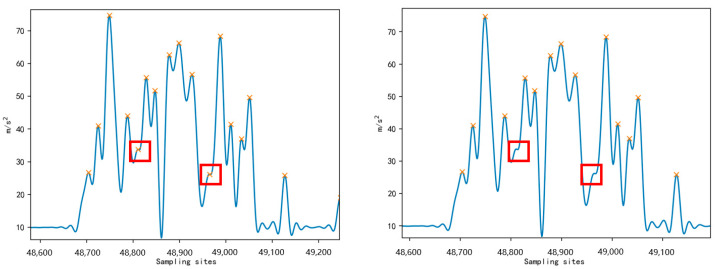
Schematic diagram of the peak and valley window detection algorithm.

Equation (2) also allows for detecting valley values within the data. The choice of the *n* value in the formula affects peak and valley values detection. If *n* is too large, some valid values may be incorrectly identified as anomalous. Conversely, if *n* is too small, some anomalous pseudo-wave peaks may be overlooked. After comparing data, it is determined that setting n to 4 is a reasonable choice for detecting peak data. Additionally, since peak and valley data always appear in pairs, valley data can effectively be detected with an *n* value of 2. The final detection result is presented in Figure 7, where the detection window successfully captured the peaks and valleys of the acceleration data while eliminating some pseudo-peaks between them. The peak detection method can calculate the steps for slow and fast walking behaviors. Specifically, when the peak value (*acc_p_*) reached the threshold condition of *thr* = 12, it was considered that the sheep completed a regular walk.

(2) $$\left\{\begin{array}{c} A={acc}_{i}-{acc}_{i-1}>0\\ \ldots \ldots \\ {acc}_{i-n}-{acc}_{i-n-1}>0\\ B={acc}_{i}-{acc}_{i+1}>0\\ \ldots \ldots \\ {acc}_{i+n}-{acc}_{i+n+1}>0\\ {acc}_{i}>thr \end{array}\right.$$

### 2.6. Behavior Detection

**Figure 8:**
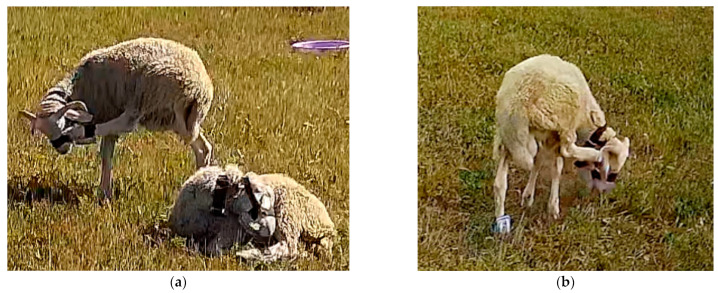
Schematic diagram of leg shaking behavior. (**a**) Scratching using the leg while wearing the device; (**b**) Scratching with the legs without wearing any device.

**Figure 9:**
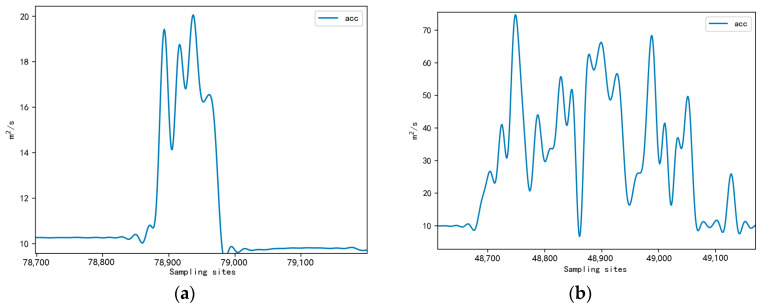
Sheep shaking legs and running waveforms. (**a**) Sheep leg shaking behavior waveform. (**b**) Sheep running behavior waveform.

Conventional peak detection algorithms can accurately count both behavioral steps when sheep walk normally or exhibit fast walking behavior. However, our experiments found that sheep exhibited some abnormal behaviors that can decrease step-counting accuracy. For example, when sheep are frightened, they will have a stress reaction and show running behavior [23], and due to insufficient data sampling rate, there were more missed steps. Sheep will show leg shaking behavior when standing or lying down, which is usually to perform scratching, and was not caused by discomfort in the legs due to the wearing of the device. The same behavior can be observed in the legs even when the device was not worn, as depicted in Figure 8, which provides a schematic representation of this behavior. Since leg-shaking behavior will lead to an increase in step counting, it is necessary to screen this behavior and remove the resulting additional steps caused. The characteristic waveforms of running and leg-shaking behaviors are shown in Figure 9. The running behavior generally had high acceleration values, and there was an emergency stop when an obstacle was encountered during the running process. The waveform showed that most peak and trough data were higher than the peak and valley data of other behaviors, and the acceleration data may show a sudden drop. The leg-shaking behavior was characterized in the waveform by a slight difference between the peak of the wave and the peak of the normal walking behavior. Still, a short time after the wave fell back and rapidly pulled up, the difference between the peak and the trough in the waveform was slight, and the waveform was unstable.

#### 2.6.1. Running Behavior Recognition

In this study, the data were collected at a frequency of 32 Hz, and through data analysis, it was concluded that the average value of sampling points for one step of normal walking for sheep was 29, meaning that sheep can walk only one step in one second. Therefore, the data when the sheep walk usually can maintain a good waveform shape at the acquisition frequency of 32 Hz, while when there is behavior like running, the acceleration data will be in a higher state all the time because the acquisition frequency is not enough. There will be multiple steps in one second, and the waveform fallback is not apparent, and so, there will be step-miss detection.

When sheep ran, the acceleration waveform peak-valley difference became small, and so, this feature can be used to complete the detection of running behavior. First, as in Equation (3), where *acc_p_* is the peak acceleration, and *acc_v_* is the valley acceleration, the start of the behavior is determined by the threshold thr1 = 30; secondly, according to the threshold thr2 = 20 and *thr*3 = 12 to determine whether the sheep is strolling or motionless and other behaviors, which is used to determine the end of the behavior; finally, the peak-valley difference detection is used to determine whether the behavior is running behavior; correspondingly, we set threshold *thr*4 = 20. Moreover, if the difference between adjacent peak and valley values in the behavior window satisfies the formula, the current behavior is determined to be running behavior.

(3) $$\left\{\begin{array}{c} {acc}_{p_{i}}>thr1\\ {acc}_{p_{i}}<thr2\;or\;{acc}_{i}<thr3\\ {acc}_{p_{i}}-{acc}_{v_{i}}<{acc}_{p_{i}}-thr4 \end{array}\right.$$

The difference of sampling points from the beginning to the end of running behavior was calculated and recorded as the running behavior window with a window size of *W*. Let R=(W ∗ K)/L, where *R* is the number of running behavior steps, *L* is the mean value of sampling points of sheep standard gait, which was taken as 29 in this paper, and *K* is the scale factor. Then, the running behavior samples were analyzed. The results of running behavior steps calculated according to different sizes of *K* values were compared with the actual manually calculated steps, and MSE (mean square error), RMSE (root mean square error), and MAE (mean absolute error) were calculated. It was finally concluded that the predicted value had the slightest error with the real value when *K* = 2.1, and the calculation results are shown in Table 2.

The following Algorithm 1 is the pseudo-code for this part of the procedure:

**Algorithm 1:** Running behavior step countingPeak: Peak data  Valley: Valley data  peak_i: Index of peak data in the original data  run: Running behavior window.  count: Running behavior steps.  1:tab1 = 0  2:tab2 = 0  3: while i < len(peak):  4:  if peak[i] > 30:  5:     tab1 = i  6:    index1 = peak_i[i]  7:    for j in range (i, len(peak)):  8:       if peak[j] < 20 or peak[j] < 12:  9:           Tab2 = j  10:         index2 = peak_i[j]  11:      For k in [I, j]:  12:         if peak[k] − valley[k] < peak[k] − 20:  13:           run = index2 − index1  14: count = (run/29) × 2.1

#### 2.6.2. Leg-Shaking Behavior Recognition

In Claudia Arcidiacono’s study, a solution for the flicking leg behavior of cows was mentioned, which used a walking period of at least three consecutive steps to address this isolated behavior [6]. However, this method was less feasible in identifying leg-shaking behavior in sheep because the normal state of sheep walking is shown as walking one step, lowering the head to graze, and so on. The waveform is shown in Figure 6, so using this method may categorize sheep’s normal walking behavior as leg shaking. Therefore, we introduced Equation (4) based on waveform feature analysis to identify leg-shaking behavior. The first three lines of Equation (4) had the same theory as the running behavior detection part above. Only the threshold size was set differently. To avoid the two behaviors interfering with each other, we added a regional peak restriction condition, i.e., when all peaks in the behavior window were smaller than the threshold thr8, the part was considered leg-shaking behavior. Based on the sample data analysis, the threshold thr8 value set to 39 was a more reasonable choice. Since the overall movement process of sheep in leg shaking behavior was not very violent compared with running behavior, the threshold values *thr*5 and *thr*7 were set to 12.

(4) $$\left\{\begin{array}{c} {acc}_{p_{i}}>thr5\\ {acc}_{i}<thr3\\ {acc}_{p_{i}}-{acc}_{v_{i}}<{acc}_{p_{i}}-thr7\\ {acc}_{p_{i}},\ldots ,{acc}_{p_{i+n}}<thr8 \end{array}\right.$$

**Figure 10:**
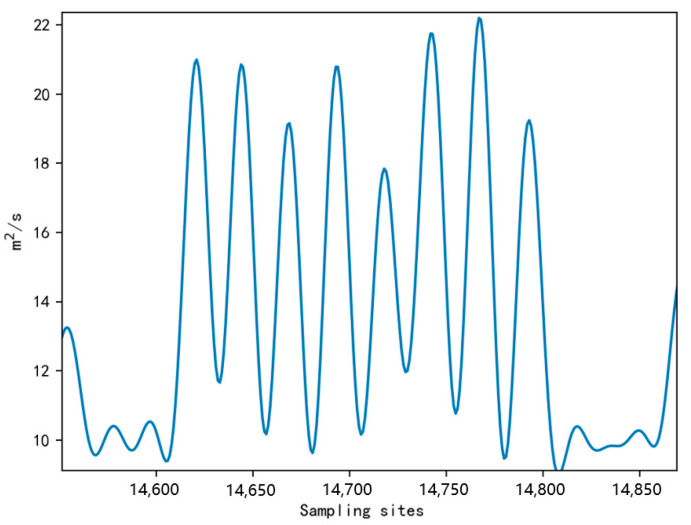
Fast walking waveform.

When counting steps for leg shaking behaviors according to the above method, we found that more other behaviors were classified as leg shaking. However, after comparing and analyzing the video and acceleration data, these misclassified behaviors were fast walking. Therefore, even if we detected that the behavior was leg shaking, we still needed to determine whether the behavior was fast walking, as shown in Figure 10 for the waveform schematic of fast walking behavior.

**Figure 11:**
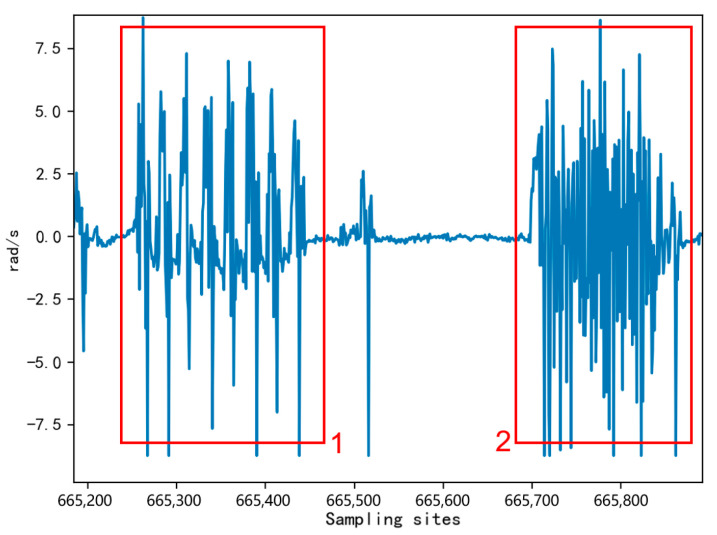
X-axis angular velocity waveforms of leg shaking and fast walking behaviors.

We analyze the acquired data for each axis of the six-axis sensor. As a result, we found more apparent features in the X-axis angular velocity that distinguish two behaviors, as shown in Figure 11, where marker 1 was the fast-walking behavior waveform and marker 2 was the leg-shaking behavior waveform.

To distinguish these two behaviors accurately, we extract the features of these two behaviors and calculated the mean, variance (var), standard deviation (std), Kurt, and skew, respectively, and used the K-means clustering algorithm to cluster the data values in three categories (standing leg shake, lying leg shake, and brisk walking behavior) after performing the feature calculation. The clustering results are referenced in Table 3.

We used each feature to predict some leg-shaking data based on the cluster analysis results. Values such as standard deviation (std) and kurtosis (Kurt) are not listed in the table for accuracy because their classification was not apparent enough. In contrast, the mean (mean), variance (var), and skewness (skew) can achieve a more precise classification. Based on the prediction results of these three features, we selected variance (var) as the differentiation condition for leg-shaking behavior and fast-walking behavior and determined the variance value as leg-shaking behavior when the variance value exceeded 10. The accuracy was calculated by Formula (5). True positive (TP) in the formula indicated that both the actual and model-predicted behavior were positive. True negative (TN) indicated that both were negative. False Positive (FP) indicated that the model-predicted category was positive, but the actual category was negative. False negative (FN) indicated that the model predicted the category as negative, but the actual category was positive. The precision, as well as the recall, were calculated by Equations (6) and (7) [13].

(5) $$Accuracy=\frac{TP+TN}{TP+FP+FN+TN}$$

(6) $$Precision=\frac{TP}{TP+FP}$$

(7) $$Recall=\frac{TP}{TP+FN}$$

The following Algorithm 2 was the pseudo-code for this part of the procedure:

**Algorithm 2:** Leg shaking behavior step countingPeak: Peak data  Valley: Valley data  xrad: x-axis angular velocity data  Count1: Shaking leg behavior steps  1: while i < len(peak):  2:  if peak[i] > 12:  3:    index1 = i  4:    for j in range (i, len(peak)):  5:      if peak[j] < 12:  6:        for k in [i, j]:  7:          if peak[k] > 39:  8:            break  9:          else  10:            if peaks[k] − Valley[k] < peaks[k] − 12:  11:              index2 = j  12:        if var(xrad[i: j]) > 10:  13:          count1 = index2 − index1

## 3. Results

To verify the algorithm’s reliability in this paper, two rams and two ewes were selected for analysis based on the experimentally collected data, and the acceleration data and video data were collected for one hour in the morning and one hour in the afternoon for four days. The step counts were performed using the algorithm designed in this paper and the peak detection algorithm, respectively, and then compared with the sheep steps counted manually by video analysis. The final statistics are shown in Table 4, and the error data were calculated by the formula (Pre-True)/(True/100). The mean value of the errors of the two algorithms was calculated, and the mean relative error of the peak detection algorithm was 17.556%. The mean relative error of the algorithm in this paper was 6.244%. This indicates that our algorithm had better stability and reliability than the peak detection algorithm.

To verify the algorithm’s performance in calculating running behavior steps and leg-shaking behavior detection, we carried out a set of experiments, and the results are shown in Table 5. In the calculation of running behavior steps, the average relative error of the typical peak detection was 19.543%, and the average relative error of this algorithm was 7.228%. Regarding the detection accuracy of leg-shaking behavior, the detection accuracy was around 92% when there were more leg-shaking behaviors. Our algorithm outperformed conventional step-counting algorithms, especially in dealing with running and leg-shaking behaviors that can interfere with step-counting.

The stationary standing behavior was used as the beginning and end of the running behavior in the manual calculation of the number of running steps because the manual did not accurately determine whether the current acceleration of the sheep reached the threshold limit. Therefore, in Table 5, the algorithm calculated the number of steps of running behavior based on the manual count of time points.

## 4. Discussion

The research in this paper aimed to develop a behavior recognition-based step-counting algorithm for low-power chips. Machine learning methods are a widely considered option for animal behavior classification using accelerometer data. However, machine learning methods are demanding regarding algorithm complexity and computational resources and may not apply to resource-limited devices such as low-power microcontrollers. This study employed a qualifying approach based on multiple thresholds to implement behavior recognition on low-power devices. This method was relatively lightweight in model size and required less computational resources and storage space while having high real-time performance and reliability. Setting multiple thresholds allowed us to classify different behaviors and achieve more accurate recognition. However, the qualification method had some limitations based on multiple thresholds. One of them was that the setting of thresholds needs to be adjusted according to different animals and behaviors, which may require some a priori knowledge or experimental validation. Therefore, although the multiple threshold-based qualification method had some advantages in resource-constrained environments, the accuracy and applicability of the algorithm still need to be considered in practical applications.

In terms of the step-counting algorithm, this study optimized the peak detection algorithm to improve the accuracy of the algorithm. The peak detection algorithm was chosen because it performed better than other algorithms [4]. In addition, implementing the algorithm was relatively simple and intuitive and did not require complex models or algorithms. The basic principle was to determine each step’s time and number of occurrences by identifying the peaks in the acceleration signal, making the step-counting calculation intuitive and easy to understand. However, the algorithm required a high sampling frequency and window size of the sensor, and if the sampling frequency was too low or the window size was not set reasonably, it will lead to inaccurate step counting. Moreover, the effect of step counting in non-smooth motion is not ideal, such as the running and leg-shaking behavior of sheep discussed in this paper. Because there are many peaks and valleys in these motion behaviors, the step length varies greatly, making it difficult for the traditional peak detection algorithm to calculate the step count accurately. For some of the behaviors with which the steps cannot be counted accurately with the traditional algorithm, the peak detection method was optimized in this paper to improve the accuracy of the step-counting algorithm. Although the optimized algorithm was designed mainly for the behavior of grazing sheep, it still improved the applicability of the step-counting algorithm to some extent. This means the same optimization strategy can be considered for similar behaviors.

Continued improvements in the accuracy of the pedometric algorithm can yield more precise data for monitoring the health of sheep, thereby enhancing their welfare. Future research could continue exploring more intelligent and adaptive algorithms to address the need for more complex behavioral patterns in step counting. This could include combining other sensor data for comprehensive analysis and using machine learning methods to build more accurate models. Indeed, this will require advances in chip technology as well as optimization of algorithms. Through these efforts, we could improve the accuracy and stability of step-counting algorithms in various behavioral scenarios, providing a more reliable solution in behavior recognition and health monitoring.

## 5. Conclusions

This study’s proposed behavior classification-based step-counting method made significant progress in sheep behavior classification and step counting. By applying multiple threshold limits to the disturbance behaviors for classification and counting steps for the corresponding behaviors, the method can calculate the daily activity steps of sheep more accurately. Compared with common peak detection step-counting methods, the proposed algorithm showed significant advantages regarding step-counting accuracy. The experimental results showed that the overall pedometer error was reduced by 11.332%, the pedometer error of running behavior was reduced by 12.315%, and the detection accuracy of leg shaking behavior reached about 92%. Although the algorithm proposed in this study achieved good results in step counting, it was still a challenging task to try to achieve perfect recognition of all the behaviors of many animals in real-life applications [24] and further improvement of the accuracy and robustness of the step-counting algorithm required consideration of more daily behavioral changes in animals. In addition, studying the relationship between step count and health status for various behaviors in grazing sheep is an important direction for future development. By understanding the behavioral changes of grazing sheep in the presence of diseases, more information can be provided for early diagnosis and monitoring of diseases.

## Figures and Tables

**Table 1:** Statistics.

Collection Date and Time	Data Format	Data Usage	
2 September 9:30–17:30	Time (yyyy/m/d h:m:s.ms) + acc (m/s^2^) + gyro (rad/s) The data’s three-axis acceleration and triaxial angular velocity units result from converting the original data.	Model Building	/
3 September 9:15–17:00		/	Algorithm validation
5 September 8:15–17:25		/	Algorithm validation
6 September 9:00–17:30		/	Algorithm validation
7 September 9:00–17:00		/	Algorithm validation
8 September 9:00–17:00		Model Building	/
10 September 9:00–17:00		Model Building	/
13 September 9:00–17:00		Model Building	/
15 September 9:45–17:00		Model Building	/
16 September 10:00–16:00		Model Building	/

**Table 2:** Difference between the results of running behavior steps calculated with different K values and the real value.

K	Prediction Error								MSE	RMSE	MAE
1.5	−2	−9	−11	−3	−6	−3	−3	−3	39.71	6.3	5.71
1.6	−1	−8	−9	−3	−5	−2	−2	−3	28.14	5.3	4.71
1.7	0	−6	−7	−2	−4	−2	−1	−2	16.29	4.04	3.43
1.8	0	−5	−5	−1	−3	−1	0	−2	9.29	3.05	2.43
1.9	1	−4	−4	0	−2	−1	0	−1	5.57	2.36	1.86
2	2	−2	−2	0	−1	0	1	0	2	1.41	1.14
2.1	2	−1	0	1	1	1	2	0	1.71	1.31	1.14
2.2	3	1	2	2	2	1	3	1	4.71	2.17	2.14
2.3	4	2	4	3	3	2	4	1	10.71	3.27	3.29
2.4	5	4	6	4	4	2	4	2	19	4.36	4.43
2.5	5	5	8	4	5	3	5	2	27.57	5.25	5.29
**True value**									**Optimal**		
	15	38	54	19	25	16	29	24	1.71	1.31	1.14

**Table 3:** Trichotomous clustering and prediction results of leg-shaking behavior.

K-Means	Mean	Var	Std	Kurt	Skew
0	0.865339	26.88633	4.680037	0.560563	0.558288
1	0.212394	7.833004	3.000012	3.21998	−0.14211
2	−0.30302	19.98162	2.094035	6.035494	−2.18545
Accuracy	0.621	0.862	/	/	0.724
Precision	0.72	0.909	/	/	0.938
Recall	0.818	0.909	/	/	0.682

**Table 4:** Step prediction results.

Date	Sheep	True	Pre1 ^1^	Pre2 ^2^	RE1 ^3^ (%)	RE2 ^4^ (%)
3 September	Ram1	147	154	155	4.76	5.44
		125	131	132	4.8	5.6
5 September	Ewe1	100	93	93	7	7
		82	158	101	92.68	23.17
6 September	Ram 2	78	88	80	12.82	2.56
		219	229	217	4.57	0.91
7 September	Ewe 2	243	238	238	2.06	2.06
		187	165	193	11.76	3.21

^1^ pre1 indicates the prediction result of the peak detection algorithm. ^2^ pre2 indicates the prediction result of this paper’s algorithm. ^3^ RE1 indicates the relative error of the peak detection calculation result. ^4^ RE2 indicates the relative error of this paper’s algorithm calculation result.

**Table 5:** Relative error of running behavior step counting and the error rate of leg shaking behavior detection.

Date	RT ^1^	R1 ^2^	R2 ^3^	S ^4^	SE ^5^	RRE1 ^6^	RRE2 ^7^	SER ^8^
3 September	56	43	60	5	1	23.21	7.14	0.2
5 September	9	8	9	25	2	11.11	0	0.08
6 September	81	65	78	12	1	19.75	3.7	0.083333
7 September	83	63	98	1	1	24.1	18.07	1

^1^ RT denotes the true value of running behavior steps. ^2^ R1 denotes the result of running behavior steps calculated by the peak detection algorithm. ^3^ R2 denotes the result of running behavior steps calculated by the algorithm in this paper. ^4^ S denotes the number of leg shaking behaviors detected. ^5^ SE denotes the number of leg shaking behavior detection errors. ^6^ RRE1 denotes the relative error of running behavior steps calculated by the peak detection algorithm. ^7^ RRE2 denotes the relative error of running behavior steps calculated by the algorithm in this paper. ^8^ SER denotes the detection error rate of leg shaking behavior.

## Data Availability

Data available on request from the authors.
